# Identification of MicroRNA as Sepsis Biomarker Based on miRNAs Regulatory Network Analysis

**DOI:** 10.1155/2014/594350

**Published:** 2014-04-06

**Authors:** Jie Huang, Zhandong Sun, Wenying Yan, Yujie Zhu, Yuxin Lin, Jiajai Chen, Bairong Shen, Jian Wang

**Affiliations:** ^1^Systems Sepsis Biology Team, Soochow University Affiliated Children's Hospital, Suzhou 215003, China; ^2^Center for Systems Biology, Soochow University, Suzhou 215006, China; ^3^Suzhou Zhengxing Translational Biomedical Informatics Ltd., Taicang 215400, China; ^4^Taicang Center for Translational Bioinformatics, Taicang 215400, China

## Abstract

Sepsis is regarded as arising from an unusual systemic response to infection but the physiopathology of sepsis remains elusive. At present, sepsis is still a fatal condition with delayed diagnosis and a poor outcome. Many biomarkers have been reported in clinical application for patients with sepsis, and claimed to improve the diagnosis and treatment. Because of the difficulty in the interpreting of clinical features of sepsis, some biomarkers do not show high sensitivity and specificity. MicroRNAs (miRNAs) are small noncoding RNAs which pair the sites in mRNAs to regulate gene expression in eukaryotes. They play a key role in inflammatory response, and have been validated to be potential sepsis biomarker recently. In the present work, we apply a miRNA regulatory network based method to identify novel microRNA biomarkers associated with the early diagnosis of sepsis. By analyzing the miRNA expression profiles and the miRNA regulatory network, we obtained novel miRNAs associated with sepsis. Pathways analysis, disease ontology analysis, and protein-protein interaction network (PIN) analysis, as well as ROC curve, were exploited to testify the reliability of the predicted miRNAs. We finally identified 8 novel miRNAs which have the potential to be sepsis biomarkers.

## 1. Introduction

Sepsis is among the common causes of death in the intensive care units' patients [1]. A well-defined reason for sepsis is the clinical syndrome resulting from the presence of both systemic inflammatory response and bacterial infection [2]. Sepsis may represent a pattern of response by the immune system to injury, with changes in the activity of thousands of endogenous mediators of inflammation, coagulation, complement, and metabolism [3]. The death toll caused by severe sepsis is of the same range as those from acute myocardial infarction [4]. The need for a timely diagnosis and accurate stratification of the severity of sepsis is no less essential, reducing mortality from sepsis [5].

Over the past decade, sepsis has been considered as a hidden public health disaster [6]. A large number of biomarkers have been proposed as candidates for sepsis diagnosis, prognosis, and therapeutic guidance. The biomarkers aim at recognizing sepsis early, so that supportive measures may be implemented as soon as possible [7, 8]. The most commonly used biomarkers of sepsis in routine clinical diagnostics are procalcitonin (PCT) and C-reactive protein(CRP) [9]. However, it is difficult to diagnose sepsis with high sensitivity and specificity at present due to the limitations of these biomarkers. MicroRNAs (miRNAs) are small noncoding RNAs that pair to sites in mRNAs to regulate gene expression in eukaryotes and play important roles in a variety of cellular functions as well as in several diseases [10–13]. Like other protein-based regulators, miRNAs have been reported as related factors to disease [14, 15]. The abnormal expression of miRNAs leads to malignant phenotypes and implicates changes in a wide array of cellular and developmental processes of disease initiation, progression, and transcriptional regulation network, such as cell proliferation, cell differentiation, apoptosis, invasion, and metastasis [10, 16, 17]. MicroRNAs are isolatable from a set of sepsis patient peripheral blood, measured by performing genome-wide profiling by microarray in leukocytes, and have been proposed to be potential sepsis biomarkers [18]. Receiver operating characteristic curves showed that miR-15a has an area under the curve of 0.858 in distinguishing sepsis patients from normal controls [19]. Serum miR-16, miR-193b*, and miR-483-5p are associated with death from sepsis and are identified as prognostic predictors of sepsis patients [20].

**Figure 1:**
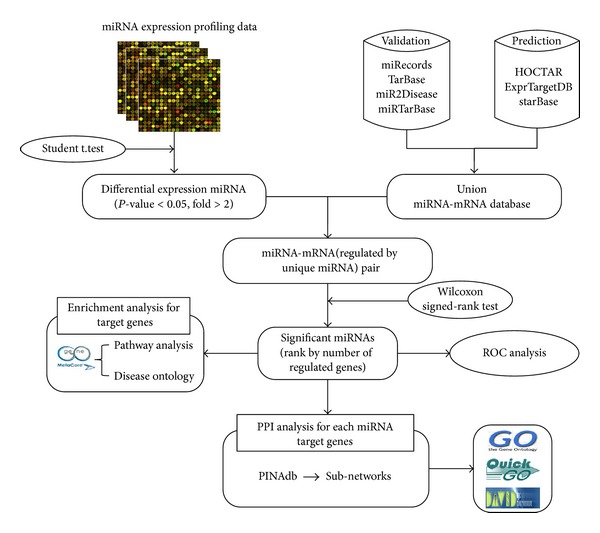
The schematic workflow in our study for identifying miRNAs as potential sepsis biomarkers.

Until now, there are many works reported to identify putative microRNA biomarkers [21–27]. Most of them detected the putative microRNA biomarkers by the analysis of differentially expressed microRNA and then verified these candidates by real-time PCR and bioinformatics analysis; they paid much attention to the multiple-multiple interaction between microRNAs and mRNAs. Few of them analyzed the substructure of microRNA-mRNA network with considering the independent regulation power of specific microRNAs. In this study, we applied an integrative analysis of miRNA regulatory networks and microarray expression profiles to identify microRNAs as sepsis biomarker. The procedure of sepsis-related miRNAs identification and analysis is illustrated in Figure 1. We previously analyzed the microRNA regulatory network [28, 29] and defined a novel out degree (NOD) to indicate the independent regulation power for an individual miRNA in the miRNA-mRNA interaction network, that is, the number of genes targeted exclusively by a specific microRNA. It means that miRNAs with larger NOD values are statistically more likely to be candidate disease biomarkers. We exploited different methods to verify the reliability of our candidate miRNA for sepsis diagnosis, and the final result reveals that these miRNAs have the potential to serve as new biomarkers for sepsis.

## 2. Materials and Methods

### 2.1. Data Collection

We conducted exhaustive search in Medline database with the key words “sepsis or severe sepsis or septic shock,” “miRNA or microRNA,” and “biomarker or marker or indicator.” Publication date (before October 31, 2013) and human studies were used as filters. We then extracted from each paper the relevant information of biomarkers, for example, microRNA name, accession number in miRBase [30], biomarker type, detection technology, study design, expression in sepsis patients, and PMID.

### 2.2. MiRNA Microarray Profiles Analysis

The miRNA expression profiles were retrieved from EBI ArrayExpress (http://www.ebi.ac.uk/arrayexpress/). The accession number is E-TABM-713 [31], produced by Vasilescu et al. The dataset contains 8 normal samples and 8 sepsis samples. We downloaded the normalized miRNA expression data directly and these profiles consist of the expression information of 556 miRNAs.

### 2.3. Statistical Methods

To identify miRNAs of interest, we adopted student* t*-test for the statistical analysis. Considering the fact that sample size is not big, we used a threshold of 0.05 for the* P* value and selected only those probe sets which showed a fold change ≥2 [26]. The miRNAs with differential expression were further ranked by their NOD values, and then Wilcoxon signed-rank test was applied to assign each miRNA a statistic significance value* P* value, indicating whether the NOD value of an individual miRNA was significantly greater than the median level of all these candidate miRNAs. We take* P* value < 0.05 as the threshold to select significant miRNAs. The ability to distinguish sepsis group and control group was characterized by the receiver operating characteristic (ROC) curve. We applied ROC analysis on the selected miRNA array data to evaluate the reliability of a biological maker or a classifier. R package epicalc [32] was used to plot the ROC curve and calculate the area under curve (AUC).

### 2.4. Union miRNA-mRNA Interactions Database

We created union miRNA-mRNA interactions for human, which combine experimentally validated targeting data and computational prediction data. The experimentally validated data were extracted from miRecords [33], TarBase [34], miR2Disease [35], and miRTarBase [36], while the computational prediction data consisted of miRNA-mRNA target pairs residing in no fewer than 2 datasets from HOCTAR [37], ExprTargetDB [38], and starBase [39]. In total, there were 32739 regulation pairs between 641 miRNAs and 7706 target genes.

### 2.5. Functional Enrichment Analysis

Herein, we mapped the genes uniquely regulated by candidate miRNAs to GeneGo database for analysis of enriched signaling pathway and disease ontology [40–42]. GeneGo database was from MetaCore. In GeneGo, hypergeometric tests were used to evaluate the statistical significance of the enriched pathways and disease. The gene ontology analysis was performed using DAVID Bioinformatics Resources 6.7 [43] and QuickGO [44].

## 3. Results and Discussion

### 3.1. Analysis of Known Sepsis miRNA Biomarker

**Figure 2:**
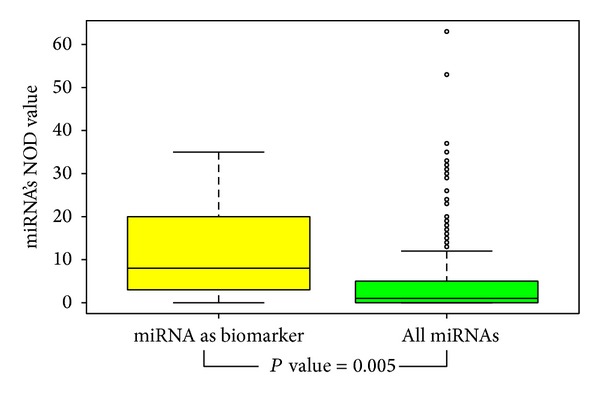
The distribution of NOD value was compared between known miRNA biomarkers and all miRNAs in database. Though we constructed miRNA-mRNA interactions network, the number of genes targeted exclusively by a specific microRNA can be computed. So each miRNA has a NOD value. Kolmogorov-Smirnov test (K-S test) was used to test whether two underlying one-dimensional probability distributions differ. The above boxplot really highlights the difference between two samples. The* P* value is 0.005 and illustrates that known miRNA biomarkers have more genes uniquely regulated by it.

Text mining in NCBI PubMed was used to identify miRNAs as sepsis biomarker. By setting the specific key words, we collated 10 miRNAs that were already proven to be helpful for diagnosis or prognosis of sepsis. To analyze common characteristics of 10 known biomarkers, the number of genes targeted exclusively by a specific microRNA in union miRNA-mRNA interactions database was conducted and we termed it as a novel out degree (NOD) to indicate the independent regulation power for an individual miRNA [28, 29]. Wilcoxon signed-rank test was applied to measure statistical significance of an individual miRNA targets count. We found that 8 of 10 (80%) known miRNA biomarkers were significantly greater than median level of all miRNAs in database; it means that miRNAs with larger NOD values are more likely to be potential sepsis biomarker. Additionally, our previous analysis of identification of cancer miRNA biomarker also suggested that miRNAs with greater independent regulation power tend more likely to be potential cancer miRNA biomarker [28, 29]. Based on this result, we can identify novel miRNA biomarker in sepsis disease. The distribution of NOD value was compared between known miRNA biomarkers and all miRNAs in database, illustrated in Figure 2.  Table 1 gives detailed information of known miRNAs biomarker which was extracted from the literature.

### 3.2. Prediction of Candidate Sepsis miRNA Biomarkers

With the result above, we exploited miRNA expression profiles to predict disease biomarker. As described in Methods, we identified 10 significantly and differentially expressed miRNAs to be candidate sepsis miRNA biomarkers from our selected miRNA expression dataset. Among these miRNAs, miR-16 [19] and miR-146a [45] have been previously reported to be sepsis biomarkers. There are some well-known miRNA biomarkers that are not presented in our list; the reason may be the heterogeneity of experimental samples and the stringent threshold we used when selecting differentially expressed miRNAs.

**Figure 3:**
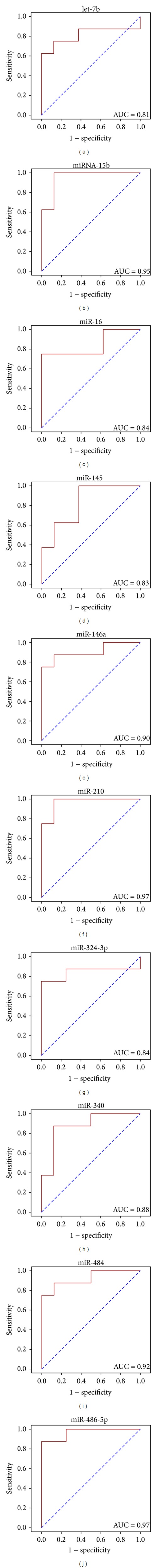
Receiver operating characteristic (ROC) curves of the 10 candidate miRNAs for their performance of diagnosis of sepsis.

The diagnostic potential of candidate miRNAs was evaluated by ROC curve analysis and the discriminatory accuracy was presented by AUC values. We found that the minimum of AUC is 0.81, the maximum is 0.97, and the average of 5 miRNAs' AUC is above 0.90. Because the property of ROC is measured as area under the curve (AUC), the ROC curve comparing sepsis patients and healthy controls provides a graphical demonstration of the superiority of candidate miRNA as sepsis marker. Finally, we plot the false positive rate (1−specificity) versus true positive rate (sensitivity) of a test (see Figure 3) for individual miRNA's ROC analysis. The detailed information on candidate miRNAs is given in Table 2.

### 3.3. Enrichment Analysis for Target Genes of the Candidate miRNAs

Previous researches have revealed that microRNAs emerged as key gene regulators in diverse biological pathways [47] and aberrant miRNA expression can contribute to human diseases [48]. It means that if a miRNA is abnormally expressed in sepsis patients, the target gene regulated by it should also change in sepsis patients. Accordingly, in order to explore the property of miRNA biomarker, we mapped the uniquely regulated genes of candidate miRNAs to GeneGo database (MetaCore) for pathway and disease ontology analysis [49, 50].

**Figure 4:**
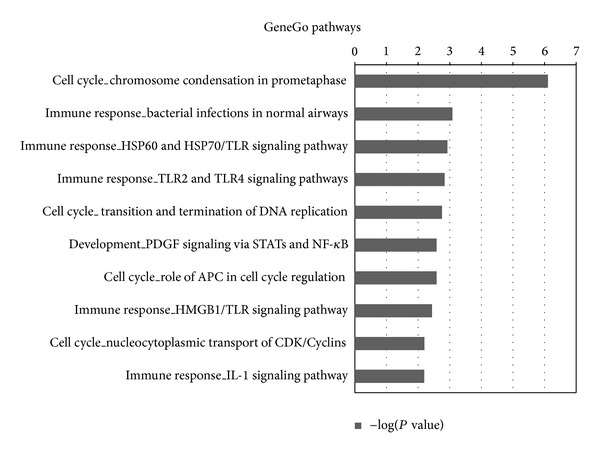
Pathway enrichment analysis for the target genes of the 10 candidate sepsis miRNA biomarkers. The uniquely regulated and targeted genes of the candidate sepsis miRNA biomarkers from our method were retrieved and annotated with analysis of pathway enrichment in GeneGo database. In total, 207 genes are uniquely regulated and targeted by the 10 candidate miRNA biomarkers. The statistical significance level* P* value was negative 10-based log transformed. Top 10 significantly enriched pathways were listed.

For pathway analysis, we retrieved 29 significantly enriched pathways (*P* value < 0.05) from GeneGo database. These pathways mapped converge on “immune response,” “cell cycle,” “apoptosis,” and “development,” which are well known to play a part in sepsis development. There are 11 pathways related to immune response; it is clear that the endotoxins of reducing sepsis interact with host cells via specific receptors on the cell surface and trigger a dysregulated immune response [51]. We also found 2 pathways for apoptosis, an important factor impacting programmed cell death and a major contributor to the pathophysiology of sepsis [52]. Among development pathways, 3 pathways about angiopoietin or cell proliferation, angiopoietin plays divergent roles in mediating inflammation and vascular quiescence [53], and cell proliferation is concomitantly observed in human severe infections [54]. The cell cycle pathways mainly contained chromosome condensation, chromosome separation, and DNA replication. The other pathways included cell adhesion, cytoskeleton remodeling, DNA damage, and metabolism. According to pathway analysis, the result well confirmed that the abnormal expression of candidate miRNAs can cause specific signaling pathway to be active in sepsis progress, and their target genes are closely related to sepsis. Therefore, our predicted candidate miRNAs are reliable for sepsis. The top 10 significant GeneGo pathways enriched with the target genes of the predicted candidate sepsis miRNAs are shown in Figure 4.

**Figure 5:**
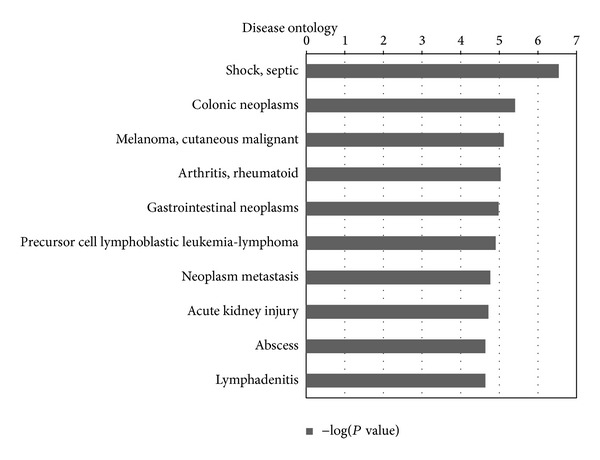
Disease ontology analysis for uniquely regulated and targeted genes of the 10 candidate sepsis miRNA biomarkers. The uniquely regulated and targeted genes of the candidate sepsis miRNA biomarkers from our method were retrieved and annotated with disease ontology analysis. In total, 207 genes are uniquely regulated and targeted by the 10 candidate miRNA biomarkers. The statistical significance level (*P* value) was negative 10-based log transformed. The top 10 significantly enriched diseases were shown.

Disease ontology is created based on the classification in medical subject headings (MeSH). Each disease in disease ontology has its corresponding biomarker gene or set of genes. After mapping the uniquely regulated and targeted genes of candidate miRNA biomarkers, we noted that the most significant disease is septic shock. Septic shock is severe sepsis plus a state of acute circulatory failure characterized by persistent arterial hypotension unexplained by other causes despite adequate volume resuscitation [55]. Based on the principle of disease ontology in GeneGo, the enriched genes are disease-related biomarkers. However, these genes are all targeted exclusively by our candidate miRNAs. This fully proves the accuracy and effectiveness of our candidate miRNAs to distinguish sepsis from healthy population. The top 10 significant disease enrichment results are listed in Figure 5.

### 3.4. The Functions of the PINs Regulated by the Candidate miRNAs

MicroRNAs implement their function by regulating their target genes, thereby directly affecting expression of their target genes at the posttranscriptional level and the related protein-protein interaction network [56]. A fundamental view is that aberrant miRNA can regulate disease progression-related biological processes [57]. If a miRNA could be the useful diagnostic marker for sepsis, the biology function of PIN regulated by it will highly relate to sepsis progression. In order to demonstrate the regulation of miRNA in sepsis crucial biological processes, we applied gene ontology analysis for miRNA regulated PIN and then validated the reliability of our candidate miRNAs.

**Figure 6:**
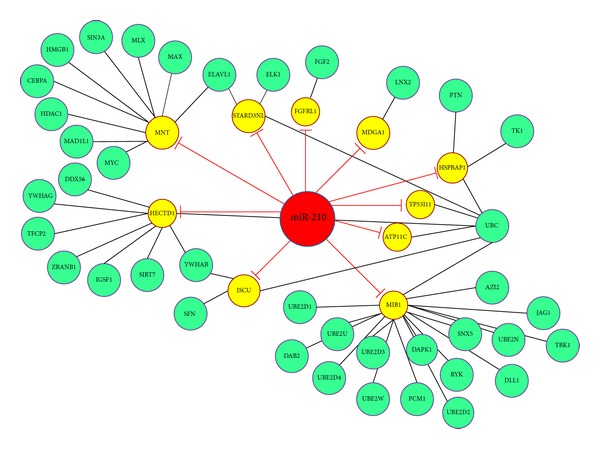
The miRNA-210 regulated protein-protein interaction network (PPIN). In this network, red node denotes the miRNA, yellow nodes denote miRNA directly targeted genes, and green nodes denote genes connected with target genes. The red lines represent a negative regulatory relationship initiated by miRNAs. The black lines represent interactions between protein and protein.

We constructed candidate miRNAs regulatory networks, containing miRNAs, genes exclusively targeted by them, and the genes directly connected to the targets. The extended network nodes were obtained by appending known interactions form the PINA database. Protein interaction network analysis (PINA) platform integrated protein-protein interaction data from six public curated databases containing 108477 binary interactions [58]. The details of 10 miRNA regulated PINs are listed in Table 3.  Figure 6 shows miR-210 regulated protein-protein interaction network, which is one of the 10 miRNA regulated PINs constructed in our work. After the construction of the 10 PINs, GO enrichment analysis was applied to elucidate their functions. We exploited DAVID to select highly significantly enriched GO terms in biology process for each miRNA regulated PIN (*P* value < 0.05). We summarized the result of GO analysis and noted that the number of nodes in individual miRNA regulated PINs was different; in addition, the number of enriched GO terms for each miRNA was also different. By extracting the common GO term of the 10 candidate miRNAs, we found that a total of 14 GO terms were included in all candidate miRNAs. The result of the GO analysis for miR-15b regulated PIN was listed in Table 4 (common GO terms for each miRNA were listed only) and result of all miRNA regulated PINs could be found in Supplementary Table S1 (see the Supplementary Material available online at available online at http://dx.doi.org/10.1155/2014/594350).

**Figure 7:**
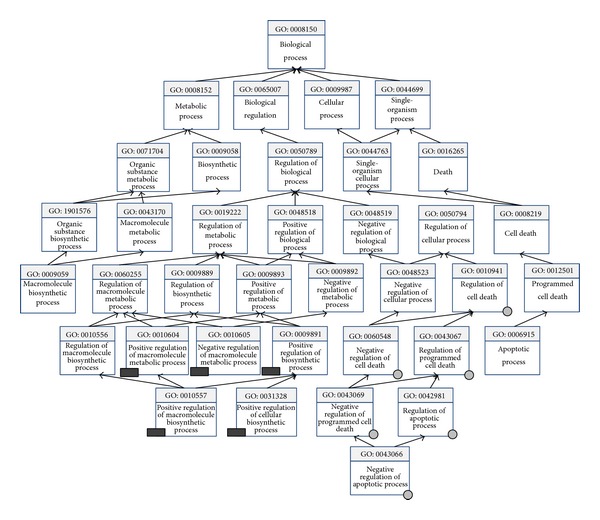
The ancestor chart for common GO terms obtained from the GO analysis of the 10 candidate miRNAs. The grey circle represents GO term related to cell death process. The black rectangle represents GO term related to macromolecule biosynthetic process. All marked GO terms are included in the common GO terms.

Further studies are needed to confirm the relationship between 14 GO terms and sepsis. The 12 of 14 terms could be divided into two processes: one is cell death and the other is macromolecule biosynthetic process. As shown in Figure 7, QuickGO was applied to build ancestor chart for the common terms. The term GO~0006916 (antiapoptosis) is the same as GO~0043066 (negative regulation of apoptosis); two other terms are related to gene expression and transcription. The pathomechanism of organ failure and death in patients with sepsis remain elusive, but programmed cell death (or apoptosis) is a key feature in sepsis, especially as it involves the lymphoid system with resulting immunoparalysis [59]. Meanwhile, macromolecule biosynthetic and metabolic process is also prominent feature in sepsis; it is related to activation and release of bacterial endotoxin, which is a macromolecule engaged in initiation of cytokine cascade [60]. The results above fully testified our candidate miRNAs by targeting specific genes to affect important biology process of sepsis progression and further illuminate the reliability of miRNA as sepsis biomarker.

## 4. Conclusions

In this study, we applied an integrative approach to identify microRNAs as sepsis biomarkers from miRNA expression profiles. Comparing with the work by Vasilescu et al., we identified 10 novel and reliable miRNA biomarkers for sepsis, supported by our pathways analysis, disease ontology analysis, and protein-protein interaction network analysis, as well as ROC curve comparison. These putative miRNA biomarkers could hopefully promote the precision diagnosis of sepsis.

## Supplementary Material

Supplementary Table S1 presents the result of gene ontology (GO) analysis for protein-protein interaction network (PIN) regulated by the sepsis miRNA biomarkers. Only the GO terms common to 10 candidate miRNAs are list.
For each GO term, the listed genes are the targets of the microRNA biomarker and their direct neighbors. The P-values are calculated based on enrichment analysis and hyper-geometric distribution.

## Figures and Tables

**Table 1:** The details of sepsis miRNA biomarkers extracted from the literature.

MicroRNA name (Hsa-)	Accession number (MIMAT)	Biomarker type	Detection technology	Study design	Expression in sepsis patients	PMID	Reference
miR-15a	0000068	Diagnosis	qRT-PCR	Serum	Up	22868808	[19]
miR-16	0000069	Diagnosis	qRT-PCR	Serum	Up	22868808	[19]
miR-122	0000421	Diagnosis	qRT-PCR	Serum	Down	23026916	[18]
miR-146a	0000449	Diagnosis	qRT-PCR	Serum	Down	20188071	[45]
miR-223	0000280	Diagnosis	qRT-PCR	Serum	Down	20188071	[45]
miR-483-5p	0004761	Prognosis	qRT-PCR	Serum	Downregulated in survivors	22719975	[20]
miR-499-5p	0002870	Diagnosis	qRT-PCR	Serum	Down	23026916	[18]
miR-574-5p	0004795	Prognosis	qRT-PCR	Serum	Upregulated in survivors	22344312	[46]
miR-150	0000451	Diagnosis	qRT-PCR	Serum	Down	19823581	[31]
miR-193b*	0004767	Prognosis	qRT-PCR	Serum	Downregulated in survivors	22719975	[20]

**Table 2:** Candidate miRNAs with outlier activity in sepsis.

MicroRNA name (Hsa-)	Accession number (MIMAT)	*P* value (sepsis patients versus controls)	Fold change (log⁡2)	NOD value	*P* value (NOD statistical significant value)	AUC value (95% CI)
let-7b	0000063	0.020	85.93	53	2.4*E* − 07	0.81
miR-16	0000069	0.030	55.79	35	3.12*E* − 07	0.84
miR-15b	0000417	0.001	192.07	33	3.82*E* − 07	0.95
miR-146a	0000449	0.002	−6.89	20	1.84*E* − 05	0.90
miR-210	0000267	0.023	1.64	15	0.0006	0.97
miR-340	0004692	0.021	−1.18	11	0.0021	0.88
miR-145	0000437	0.021	13.03	11	0.0021	0.83
miR-484	0002174	0.002	3.74	11	0.0021	0.92
miR-324-3p	0000762	0.021	2.45	10	0.0041	0.84
miR-486-5p	0002177	0.019	102.49	8	0.0151	0.97

**Table 3:** Summary of constructed 10 miRNA regulated PINs. N0: gene was included in PINA database; N1: the extended subnetwork of N0 gene directly connected to N0 gene; N2: the total genes of miRNA regulated subnetwork.

MicroRNA name (Hsa-)	Accession number (MIMAT)	NOD count	N0 count	N1 count	N2 count
let-7b	0000063	53	42	424	466
miR-15b	0000417	33	26	201	227
miR-16	0000069	35	28	384	412
miR-145	0000437	11	8	256	264
miR-146a	0000449	20	13	202	215
miR-210	0000267	15	10	39	49
miR-324-3p	0000762	10	10	121	131
miR-340	0004692	11	9	124	133
miR-484	0002174	11	11	246	257
miR-486-5p	0002177	8	6	26	32

**Table 4:** GO analysis results of miR-15b regulated PIN. The common GO terms for miR-15b were listed.

MIMAT0000417 (Hsa-miR-15b)		
GO term	Genes	*P* value
GO:0006916~antiapoptosis	BFAR, HSP90B1, GSK3B, BCL2, HIPK3, TGFBR1, NPM1, UBC, SERPINB2, FAIM3, BCL2L1, HSPA5	2.96*E* − 04

GO:0009891~positive regulation of biosynthetic process	DVL3, HRAS, THRB, GRIP1, PCBD1, RXRB, RXRA, TGFBR1, PPARG, DDX5, CALR, POT1, SREBF2, ATXN1, MAPK1, MEIS2, PSMC5, NCOA2, HNF4A, ATXN7, NPM1, UBC, YAP1	8.10*E* − 04

GO:0010557~positive regulation of macromolecule biosynthetic process	DVL3, HRAS, THRB, GRIP1, PCBD1, RXRB, RXRA, TGFBR1, PPARG, DDX5, CALR, POT1, SREBF2, ATXN1, MAPK1, MEIS2, PSMC5, NCOA2, HNF4A, ATXN7, UBC, YAP1	8.92*E* − 04

GO:0010604~positive regulation of macromolecule metabolic process	HRAS, THRB, GRIP1, PPARG, PSMD1, PSMD2, PSMD3, H2AFX, PSMD4, YAP1, PSMD6, PSMD7, PRKCA, PCBD1, RXRB, RXRA, PSMA2, UBE2N, MAPK1, NCOA2, HNF4A, PSMA6, PSMA3, UBC, MDM2, CALR, POT1, PIN1, PSMB5, MEIS2, BCL2, UBE2D1, DVL3, TGFBR1, DDX5, FURIN, SREBF2, ATXN1, PSMC6, PSMD14, PSMD13, PSMC5, PSMD12, PSMC4, PSMC3, PSMD11, PSMD10, ATXN7, PSMC2, PSMC1	1.54*E* − 16

GO:0010605~negative regulation of macromolecule metabolic process	THRB, TSG101, PPARG, BCL2L1, TERF2IP, CALR, POT1, PSMB5, MEIS2, NPM1, PSMD1, PSMD2, PSMD3, PSMD4, UBE2D1, PSMD6, PSMD7, PRKCA, RXRA, ZNF24, UBE2I, FURIN, CDK5, SIRT3, PSMA2, ATXN1, PSMD14, PSMC6, PSMD13, NCOA2, PSMC5, PSMA6, HNF4A, PSMD12, PSMC4, PSMC3, PSMD11, PSMD10, PSMC2, PSMA3, PSMC1, UBC, BUB1B, MDM2, FABP4, SMURF2	3.72*E* − 16

GO:0010628~positive regulation of gene expression	DVL3, THRB, GRIP1, RXRB, PCBD1, RXRA, TGFBR1, PPARG, DDX5, SREBF2, ATXN1, MAPK1, MEIS2, PSMC5, NCOA2, HNF4A, ATXN7, UBC, YAP1	0.0031

GO:0010941~regulation of cell death	HRAS, BCAR1, BCL2L1, CALR, ITSN1, DYNLL1, BCL2, SOS1, CASP8, RAC1, NPM1, POU4F1, HSPA5, PRKCA, VAV3, TP53BP2, TGFBR1, TMBIM6, RXRA, ACTN1, ACTN2, FURIN, VAV1, CDK5, CASP10, MAPK1, BFAR, HSP90B1, PSMC5, GSK3B, HIPK3, UBC, SERPINB2, ERN1, FAIM3, MAPK8, CACNA1A	4.80*E* − 09

GO:0031328~positive regulation of cellular biosynthetic process	DVL3, HRAS, THRB, GRIP1, PCBD1, RXRB, RXRA, TGFBR1, PPARG, DDX5, CALR, POT1, SREBF2, ATXN1, MAPK1, MEIS2, PSMC5, NCOA2, HNF4A, ATXN7, NPM1, UBC, YAP1	6.69*E* − 04

GO:0042981~regulation of apoptosis	HRAS, BCAR1, BCL2L1, CALR, ITSN1, DYNLL1, BCL2, SOS1, CASP8, RAC1, NPM1, POU4F1, HSPA5, PRKCA, VAV3, TP53BP2, TGFBR1, TMBIM6, RXRA, ACTN1, ACTN2, FURIN, VAV1, CDK5, CASP10, MAPK1, BFAR, HSP90B1, GSK3B, HIPK3, UBC, SERPINB2, ERN1, FAIM3, MAPK8, CACNA1A	1.18*E* − 08

GO:0043066~negative regulation of apoptosis	HRAS, TMBIM6, TGFBR1, BCL2L1, ITSN1, FURIN, BFAR, HSP90B1, GSK3B, HIPK3, BCL2, NPM1, UBC, SERPINB2, FAIM3, MAPK8, HSPA5, CACNA1A	2.61*E* − 05

GO:0043067~regulation of programmed cell death	HRAS, BCAR1, BCL2L1, CALR, ITSN1, DYNLL1, BCL2, SOS1, CASP8, RAC1, NPM1, POU4F1, HSPA5, PRKCA, VAV3, TP53BP2, TGFBR1, TMBIM6, RXRA, ACTN1, ACTN2, FURIN, VAV1, CDK5, CASP10, MAPK1, BFAR, HSP90B1, PSMC5, GSK3B, HIPK3, UBC, SERPINB2, ERN1, FAIM3, MAPK8, CACNA1A	4.35*E* − 09

GO:0043069~negative regulation of programmed cell death	HRAS, TMBIM6, TGFBR1, BCL2L1, ITSN1, FURIN, BFAR, HSP90B1, PSMC5, GSK3B, HIPK3, BCL2, NPM1, UBC, SERPINB2, FAIM3, MAPK8, HSPA5, CACNA1A	8.38*E* − 06

GO:0045941~positive regulation of transcription	DVL3, THRB, GRIP1, RXRB, PCBD1, RXRA, TGFBR1, PPARG, DDX5, SREBF2, ATXN1, MAPK1, MEIS2, PSMC5, NCOA2, HNF4A, ATXN7, UBC, YAP1	0.0022

GO:0060548~negative regulation of cell death	HRAS, TMBIM6, TGFBR1, BCL2L1, ITSN1, FURIN, BFAR, HSP90B1, PSMC5, GSK3B, HIPK3, BCL2, NPM1, UBC, SERPINB2, FAIM3, MAPK8, HSPA5, CACNA1A	8.76*E* − 06
