# Supplementary material for: Identification of MicroRNA as Sepsis Biomarker Based on miRNAs Regulatory Network Analysis
Source: Biomed Res Int. 2014 Apr 6;2014:594350. doi: 10.1155/2014/594350 (PMC3997997; doi:10.1155/2014/594350)
Supplement: Supplementary file 1 — Supplementary Table S1 presents the result of gene ontology (GO) analysis for protein-protein interaction network (PIN) regulated by the sepsis miRNA biomarkers. Only the GO terms common to 10 candidate miRNAs are list. For each GO term, the listed genes are the targets of the microRNA biomarker and their direct neighbors. The P-values are calculated based on enrichment analysis and hyper-geometric distribution. [file 594350.f1.pdf]

Table S1:

MIMAT0000063(has-let-7b)		
**GO-term**	**Genes**	***P-value***
GO:0006916~anti-apoptosis	STAMBP, IRAK1, VHL, RELA, GSK3B, STAT5A, STAT5B, UBC, NFKBIA, TRAF6, MYC, TAX1BP1	0.0296
GO:0009891~positive regulation of biosynthetic process	NIF3L1, PDGFB, PDGFA, STAT5A, STAT5B, TGFB3, NFKBIA, PAX3, NR1H2, SMARCD1, PDGFC, MYOG, TCEA1, SHC1, ERCC3, SUPT5H, TRAF6, MYC, EIF2B5, IRAK1, HSP90AA1, MYO6, VHL, PCBD1, RELA, ILF3, SIRT7, KAT5, DDX5, STAT3, CDK2, GTF2H1, ATXN1, HDAC4, ATF4, EP300, MED17, GTF2F1, GTF2F2, SMARCC2, UBC, PDGFRA, CAND1	1.85E-06
GO:0010557~positive regulation of macromolecule biosynthetic process	NIF3L1, PDGFB, PDGFA, STAT5A, STAT5B, TGFB3, NFKBIA, PAX3, NR1H2, SMARCD1, PDGFC, MYOG, TCEA1, SHC1, ERCC3, SUPT5H, TRAF6, MYC, EIF2B5, IRAK1, MYO6, VHL, PCBD1, RELA, ILF3, SIRT7, KAT5, DDX5, STAT3, CDK2, GTF2H1, ATXN1, HDAC4, ATF4, EP300, MED17, GTF2F1, GTF2F2, SMARCC2, UBC, PDGFRA, CAND1	9.78E-07
GO:0010604~positive regulation of macromolecule metabolic process	NIF3L1, PDGFB, PDGFA, STAT5A, BTRC, STAT5B, TGFB3, PAX3, SMARCD1, SHC1, PDGFC, ITCH, SUPT5H, MYC, CUL1, EIF2B5, IRAK1, MYO6, PCBD1, RELA, CDK2, GTF2H1, PSMA1, CCND1, EP300, PSME1, CCND3, PIAS4, CCND2, PIAS3, MED17, PSMA3, UBC, PDGFRA, CAND1, IFT88, CAV1, NFKBIA, KIT, PIN1, NR1H2, RB1CC1, TCEA1, MYOG, TRAF6, ERCC3, DNMT3B, VHL, ILF3, SIRT7, DDX5, KAT5, STAT3, ATXN1, HDAC4, ATF4, NEDD4, PLK1, GTF2F1, GTF2F2, SMARCC2, BRE	8.76E-12
GO:0010605~negative regulation of macromolecule metabolic process	CAV1, SNX6, TSG101, TH1L, TGFB3, MLH1, ITGB3, PAX3, DAXX, LIN28B, VPS72, NR1H2, SUMO1, ATN1, EIF3E, PPP2CA, ITGAV, EED, DDX20, SUPT5H, APOM, MYC, DNMT3B, EIF2B5, VHL, RELA, ILF3, SIRT7, KAT5, SIRT1, STAT3, COBRA1, ATXN1, HDAC4, PSMA1, PSME1, PIAS4, NEDD4, PSMA3, SMARCC2, UBC, HDAC8, EIF2C3, EIF2C4	3.05E-06
GO:0010628~positive regulation of gene expression	NIF3L1, STAT5A, STAT5B, TGFB3, NFKBIA, PAX3, KIT, NR1H2, SMARCD1, MYOG, TCEA1, ERCC3, SUPT5H, MYC, DNMT3B, IRAK1, MYO6, VHL, PCBD1, RELA, ILF3, SIRT7, KAT5, DDX5, CDK2, STAT3, GTF2H1, ATXN1, HDAC4, ATF4, EP300, MED17, GTF2F1, GTF2F2, SMARCC2, UBC, CAND1	6.03E-06
GO:0010941~regulation of cell death	TRAF2, STAT5A, FASTK, STAT5B, TGFB3, NFKBIA, MLH1, INTS1, KIT, DAXX, PTEN, SRC, SMNDC1, CUL3, CUL5, CASP3, RB1CC1, PPP2CA, DDX20, ERCC3, TRAF6, MYC, TRAF4, CUL1, STAMBP, IRAK1, VHL, RELA, WRN, STAT1, SIRT1, TAX1BP1, CDKN1A, GSK3B, IKBKG, UBC, SMPD1, ABL1	0.0023
GO:0031328~positive regulation of cellular biosynthetic process	NIF3L1, PDGFB, PDGFA, STAT5A, STAT5B, TGFB3, NFKBIA, PAX3, NR1H2, SMARCD1, PDGFC, MYOG, TCEA1, SHC1, ERCC3, SUPT5H, TRAF6, MYC, EIF2B5, IRAK1, HSP90AA1, MYO6, VHL, PCBD1, RELA, ILF3, SIRT7, KAT5, DDX5, STAT3, CDK2, GTF2H1, ATXN1, HDAC4, ATF4, EP300, MED17, GTF2F1, GTF2F2, SMARCC2, UBC, PDGFRA, CAND1	1.28E-06
GO:0042981~regulation of apoptosis	TRAF2, STAT5A, FASTK, STAT5B, TGFB3, NFKBIA, MLH1, INTS1, DAXX, PTEN, SRC, SMNDC1, CUL3, CUL5, CASP3, RB1CC1, PPP2CA, DDX20, ERCC3, TRAF6, MYC, TRAF4, CUL1, STAMBP, IRAK1, VHL, RELA, WRN, STAT1, SIRT1, TAX1BP1, CDKN1A, GSK3B, IKBKG, UBC, SMPD1, ABL1	0.0033
GO:0043066~negative regulation of apoptosis	STAMBP, IRAK1, VHL, STAT5A, RELA, STAT5B, TGFB3, NFKBIA, INTS1, PTEN, TAX1BP1, CDKN1A, CASP3, GSK3B, RB1CC1, UBC, TRAF6, MYC	0.0206
GO:0043067~regulation of programmed cell death	TRAF2, STAT5A, FASTK, STAT5B, TGFB3, NFKBIA, MLH1, INTS1, KIT, DAXX, PTEN, SRC, SMNDC1, CUL3, CUL5, CASP3, RB1CC1, PPP2CA, DDX20, ERCC3, TRAF6, MYC, TRAF4, CUL1, STAMBP, IRAK1, VHL, RELA, WRN, STAT1, SIRT1, TAX1BP1, CDKN1A, GSK3B, IKBKG, UBC, SMPD1, ABL1	0.0021
GO:0043069~negative regulation of programmed cell death	STAMBP, IRAK1, VHL, STAT5A, RELA, STAT5B, TGFB3, NFKBIA, INTS1, KIT, PTEN, TAX1BP1, CDKN1A, CASP3, GSK3B, RB1CC1, UBC, TRAF6, MYC	0.0118
GO:0045941~positive regulation of transcription	NIF3L1, STAT5A, STAT5B, TGFB3, NFKBIA, PAX3, NR1H2, SMARCD1, MYOG, TCEA1, ERCC3, SUPT5H, MYC, IRAK1, MYO6, VHL, PCBD1, RELA, ILF3, SIRT7, DDX5, KAT5, CDK2, STAT3, GTF2H1, ATXN1, HDAC4, ATF4, EP300, MED17, GTF2F1, GTF2F2, SMARCC2, UBC, CAND1	1.97E-05
GO:0060548~negative regulation of cell death	STAMBP, IRAK1, VHL, STAT5A, RELA, STAT5B, TGFB3, NFKBIA, INTS1, KIT, PTEN, TAX1BP1, CDKN1A, CASP3, GSK3B, RB1CC1, UBC, TRAF6, MYC	0.0121

Table S1. *Cont.*

MIMAT0000069(Hsa-miR-16)		
**GO-term**	**Genes**	**P-value**
GO:0006916~anti-apoptosis	MEF2C, PRKCZ, YWHAZ, STAT5A, SNCA, FOXO1, HSPA1B, AKT1S1, MYD88, SQSTM1, BAG3, NPM1, TGM2, PIK3CA, TRAF6, MYC, CDK1, IRAK1, TGFBR1, PRKCI, ESR1, BIRC6, MALT1, BIRC5, ESR2, UBC, FOXC2, UBB, UBA52	1.38E-12
GO:0009891~positive regulation of biosynthetic process	E2F1, MEF2C, MMS19, STAT5A, FOXO1, PAWR, CNOT7, CBFB, CCNE1, USP16, NR2F2, CCNA2, MYC, BCL10, IRAK1, AR, HSP90AA1, RB1, CDK7, ESR2, CDK4, HMGA1, GTF2H1, MAPK1, NCOA3, BPTF, UBC, FOXC2, CAND1, NFE2L2, UBB, UBA52, POT1, TNFRSF1A, SQSTM1, NPM1, TRAF6, RUNX2, ERCC2, UCN, TGFBR1, SMAD4, SMAD3, SMAD2, SMAD1, SIRT7, KAT5, TP73, BRCA1, HDAC5, ATF4, MAPK14, JAK3, MTOR, HDAC6	1.27E-12
GO:0010557~positive regulation of macromolecule biosynthetic process	E2F1, MEF2C, MMS19, STAT5A, FOXO1, PAWR, CNOT7, CBFB, CCNE1, USP16, NR2F2, CCNA2, MYC, BCL10, IRAK1, AR, RB1, CDK7, ESR2, CDK4, HMGA1, GTF2H1, MAPK1, NCOA3, BPTF, UBC, FOXC2, CAND1, NFE2L2, UBB, UBA52, POT1, TNFRSF1A, SQSTM1, TRAF6, RUNX2, ERCC2, UCN, TGFBR1, SMAD4, SMAD3, SMAD2, SMAD1, SIRT7, KAT5, TP73, BRCA1, HDAC5, ATF4, MAPK14, JAK3, MTOR, HDAC6	1.45E-12
GO:0010604~positive regulation of macromolecule metabolic process	MMS19, E2F1, MEF2C, STAT5A, SNCA, FOXO1, PAWR, CNOT7, CBFB, CCNE1, ATG7, PSMD4, H2AFX, MLST8, NR2F2, USP16, CCNA2, MYC, CUL1, CDK1, IRAK1, BCL10, AR, LYN, ESR1, RB1, ESR2, CDK7, CDK4, HMGA1, GTF2H1, MAPK1, CCND1, BPTF, NCOA3, UBC, FOXC2, CAND1, UBB, NFE2L2, UBA52, PML, POT1, PIN1, TNFRSF1A, SQSTM1, TRAF6, RUNX2, TINF2, ERCC2, UCN, MAP2K1, TGFBR1, SMAD4, SMAD3, SMAD2, SIRT7, SMAD1, KAT5, TP73, BRCA1, CCNB1, HDAC5, ATF4, PLK1, MAPK14, PSMC2, BRE, JAK3, SMURF1, MTOR, TNK2, HDAC6	1.09E-18
GO:0010605~negative regulation of macromolecule metabolic process	MEF2C, E2F1, TSG101, SNCA, MLH1, DMAP1, PAWR, CDT1, CDKN2A, PSMD4, NR2F2, MYC, KHDRBS1, IBTK, PPP2R1A, TLE1, RB1, MBD2, BPTF, UBC, MDM4, UBB, CUX1, UBA52, PRKCZ, ENPP1, TH1L, PML, GIPC1, ZBTB16, POT1, ACD, NPM1, RUNX2, TINF2, SMAD4, SMAD3, BRCA2, SMAD2, SMAD1, SIRT7, KAT5, BRCA1, HDAC5, TSC1, PHB2, PSMC2, IRF8, YWHAQ, SMURF2, HDAC6	1.01E-09
GO:0010628~positive regulation of gene expression	MEF2C, E2F1, MMS19, STAT5A, FOXO1, CNOT7, CBFB, CCNE1, TNFRSF1A, SQSTM1, NR2F2, USP16, RUNX2, MYC, CCNA2, ERCC2, BCL10, IRAK1, AR, TGFBR1, ESR1, SMAD4, SMAD3, SMAD2, RB1, CDK7, SMAD1, SIRT7, ESR2, KAT5, HMGA1, BRCA1, TP73, GTF2H1, HDAC5, MAPK1, ATF4, NCOA3, BPTF, MAPK14, UBC, FOXC2, CAND1, NFE2L2, JAK3, UBB, UBA52	3.78E-11
GO:0010941~regulation of cell death	MEF2C, STAT5A, SNCA, MLH1, FOXO1, PAWR, ITSN1, CUL3, CUL2, CDKN2A, MYD88, BAG3, ILK, CASP8, PIK3CA, TOP2A, MYC, CUL1, BCL10, IRAK1, CDK1, PPP2R1A, ESR1, PRKCI, ESR2, ECT2, MAPK1, TNFRSF9, KRT18, RIPK1, LCK, UBC, FOXC2, UBB, UBA52, TRAF1, PRKCZ, TRAF2, YWHAZ, PML, HSPA1B, SFN, ZBTB16, AKT1S1, SQSTM1, NPM1, PYCARD, TGM2, TRAF6, PHLDA3, ERCC2, UCN, TP53BP2, TGFBR1, SMAD3, BIRC6, BRCA2, BIRC5, MALT1, TP73, BRCA1, CDKN1A, VCP, NTRK1, IKBKG, ABL1, HDAC6, DNM2	6.44E-17
GO:0031328~positive regulation of cellular biosynthetic process	E2F1, MEF2C, MMS19, STAT5A, FOXO1, PAWR, CNOT7, CBFB, CCNE1, USP16, NR2F2, CCNA2, MYC, BCL10, IRAK1, AR, HSP90AA1, RB1, CDK7, ESR2, CDK4, HMGA1, GTF2H1, MAPK1, NCOA3, BPTF, UBC, FOXC2, CAND1, NFE2L2, UBB, UBA52, POT1, TNFRSF1A, SQSTM1, NPM1, TRAF6, RUNX2, ERCC2, UCN, TGFBR1, SMAD4, SMAD3, SMAD2, SMAD1, SIRT7, KAT5, TP73, BRCA1, HDAC5, ATF4, MAPK14, JAK3, MTOR	2.49E-12
GO:0042981~regulation of apoptosis	MEF2C, STAT5A, SNCA, MLH1, FOXO1, PAWR, ITSN1, CUL3, CUL2, CDKN2A, MYD88, BAG3, ILK, CASP8, PIK3CA, TOP2A, MYC, CUL1, BCL10, IRAK1, CDK1, PPP2R1A, ESR1, PRKCI, ESR2, ECT2, MAPK1, TNFRSF9, KRT18, RIPK1, LCK, UBC, FOXC2, UBB, UBA52, TRAF1, PRKCZ, TRAF2, YWHAZ, PML, HSPA1B, SFN, ZBTB16, AKT1S1, SQSTM1, NPM1, PYCARD, TGM2, TRAF6, PHLDA3, ERCC2, UCN, TP53BP2, TGFBR1, SMAD3, BIRC6, BRCA2, BIRC5, MALT1, TP73, BRCA1, CDKN1A, VCP, NTRK1, IKBKG, ABL1, HDAC6, DNM2	3.23E-17
GO:0043066~negative regulation of apoptosis	MEF2C, PRKCZ, YWHAZ, STAT5A, SNCA, FOXO1, HSPA1B, ITSN1, MYD88, AKT1S1, SQSTM1, BAG3, ILK, NPM1, TGM2, PIK3CA, TRAF6, MYC, ERCC2, CDK1, IRAK1, BCL10, TGFBR1, PRKCI, ESR1, BIRC6, SMAD3, MALT1, BIRC5, ESR2, TP73, CDKN1A, KRT18, NTRK1, UBC, FOXC2, UBB, UBA52	1.21E-12

GO:0043067~regulation of programmed cell death	MEF2C, STAT5A, SNCA, MLH1, FOXO1, PAWR, ITSN1, CUL3, CUL2, CDKN2A, MYD88, BAG3, ILK, CASP8, PIK3CA, TOP2A, MYC, CUL1, BCL10, IRAK1, CDK1, PPP2R1A, ESR1, PRKCI, ESR2, ECT2, MAPK1, TNFRSF9, KRT18, RIPK1, LCK, UBC, FOXC2, UBB, UBA52, TRAF1, PRKCZ, TRAF2, YWHAZ, PML, HSPA1B, SFN, ZBTB16, AKT1S1, SQSTM1, NPM1, PYCARD, TGM2, TRAF6, PHLDA3, ERCC2, UCN, TP53BP2, TGFBR1, SMAD3, BIRC6, BRCA2, BIRC5, MALT1, TP73, BRCA1, CDKN1A, VCP, NTRK1, IKBKG, ABL1, HDAC6, DNM2	5.34E-17
GO:0043069~negative regulation of programmed cell death	MEF2C, PRKCZ, YWHAZ, STAT5A, SNCA, FOXO1, HSPA1B, ITSN1, MYD88, AKT1S1, SQSTM1, BAG3, ILK, NPM1, TGM2, PIK3CA, TRAF6, MYC, ERCC2, CDK1, IRAK1, BCL10, TGFBR1, PRKCI, ESR1, BIRC6, SMAD3, MALT1, BIRC5, ESR2, TP73, CDKN1A, KRT18, NTRK1, UBC, FOXC2, UBB, UBA52	1.84E-12
GO:0045941~positive regulation of transcription	MEF2C, E2F1, MMS19, STAT5A, FOXO1, CNOT7, CBFB, CCNE1, TNFRSF1A, SQSTM1, NR2F2, USP16, RUNX2, MYC, CCNA2, ERCC2, BCL10, IRAK1, AR, TGFBR1, SMAD4, SMAD3, SMAD2, RB1, CDK7, SMAD1, SIRT7, ESR2, KAT5, HMGA1, BRCA1, TP73, GTF2H1, HDAC5, MAPK1, ATF4, NCOA3, BPTF, MAPK14, UBC, FOXC2, CAND1, NFE2L2, JAK3, UBB, UBA52	4.92E-11
GO:0060548~negative regulation of cell death	MEF2C, PRKCZ, YWHAZ, STAT5A, SNCA, FOXO1, HSPA1B, ITSN1, MYD88, AKT1S1, SQSTM1, BAG3, ILK, NPM1, TGM2, PIK3CA, TRAF6, MYC, ERCC2, CDK1, IRAK1, BCL10, TGFBR1, PRKCI, ESR1, BIRC6, SMAD3, MALT1, BIRC5, ESR2, TP73, CDKN1A, KRT18, NTRK1, UBC, FOXC2, UBB, UBA52	2.00E-12

Table S1. *Cont.*

MIMAT0000437(hsa-miR-145)		
**GO-term**	**Genes**	**P-value**
GO:0006916~anti-apoptosis	AKT1, HMGB1, IRAK1, IL2RB, YWHAZ, MYD88, RELA, STAT5A, TGFBR1, STAT5B, UBC, TRAF6	0.0011
GO:0009891~positive regulation of biosynthetic process	E2F1, HSP90AB1, HMGB2, STAT5A, SOX2, STAT5B, TLR4, SOX8, AKT1, TNFRSF1A, FOS, APP, TICAM1, BCL3, TCF4, NR2F2, TRAF6, SYK, EGFR, IRAK1, SOX10, AR, TGFBR1, RELA, TP53BP1, RXRA, TRIM28, CREBBP, SMAD2, BRCA1, STAT3, HDAC4, MNAT1, SALL4, YWHAH, EP300, MAPK14, ETS2, UBC, PDGFRA, IRF1, JAK2, MTOR, PIAS1, SMARCA2, SMARCA4	2.89E-14
GO:0010557~positive regulation of macromolecule biosynthetic process	E2F1, HMGB2, STAT5A, SOX2, STAT5B, TLR4, SOX8, AKT1, TNFRSF1A, FOS, APP, TICAM1, BCL3, TCF4, NR2F2, TRAF6, SYK, SOX10, IRAK1, AR, TGFBR1, RELA, TP53BP1, RXRA, TRIM28, CREBBP, SMAD2, BRCA1, STAT3, HDAC4, MNAT1, SALL4, YWHAH, EP300, MAPK14, ETS2, UBC, PDGFRA, IRF1, MTOR, PIAS1, SMARCA2, SMARCA4	3.16E-13
GO:0010604~positive regulation of macromolecule metabolic process	E2F1, IL6ST, STAT5A, STAT5B, TLR4, AKT1, FOS, APP, TICAM1, NR2F2, SYK, IRAK1, SOX10, AR, RELA, RXRA, EP300, UBC, PDGFRA, PIAS1, SMARCA2, SMARCA4, HMGB2, SOX2, KIT, SOX8, TNFRSF1A, BCL3, TRAF6, TCF4, DNAJA3, IL2RB, TGFBR1, TP53BP1, CREBBP, TRIM28, SMAD2, BRCA1, STAT3, MNAT1, HDAC4, YWHAH, SALL4, PSMC3, MAPK14, ETS2, PSMD10, IRF1, ADRA1B, JAK2, SMURF1, MTOR	1.19E-14
GO:0010605~negative regulation of macromolecule metabolic process	E2F1, HSP90AB1, HMGB1, HMGB2, SOX2, BAK1, VDR, SUMO1, SORBS3, SET, WWP2, PRKRA, BCL3, TCF4, NR2F2, ZNF281, RELA, RXRA, SOCS1, TRIM28, YWHAB, SMAD2, UBE2I, NR0B1, PRKCD, BRCA1, STAT3, PURA, HDAC4, SALL4, PSMC3, PSMD10, RNF2, YWHAQ, UBC, ADRA1B, IRF2, SMARCA2, EIF2AK4, SMARCA4	7.22E-10
GO:0010628~positive regulation of gene expression	E2F1, HMGB2, STAT5A, SOX2, STAT5B, KIT, SOX8, FOS, TNFRSF1A, APP, BCL3, TCF4, NR2F2, SOX10, IRAK1, IL2RB, AR, TGFBR1, RELA, TP53BP1, RXRA, CREBBP, TRIM28, SMAD2, BRCA1, STAT3, HDAC4, MNAT1, SALL4, YWHAH, EP300, MAPK14, ETS2, UBC, IRF1, PIAS1, SMARCA2, SMARCA4	1.26E-11
GO:0010941~regulation of cell death	HMGB1, YWHAZ, STAT5A, ERBB2, STAT5B, TLR2, TLR4, CHEK2, KIT, SRC, BTK, CTNNBL1, AKT1, VDR, BAK1, APP, CASP3, MYD88, RAC1, PRKRA, TICAM1, RHOA, BCL3, CASP1, TRAF6, DNAJA3, EGFR, IRAK1, IL2RB, ACTN4, DFFA, TGFBR1, RELA, RXRA, YWHAB, CIDEB, FADD, PRKCE, STAT1, BRCA1, TRADD, MNAT1, LCK, UBC, JAK2	2.88E-11
GO:0031328~positive regulation of cellular biosynthetic process	E2F1, HSP90AB1, HMGB2, STAT5A, SOX2, STAT5B, TLR4, SOX8, AKT1, TNFRSF1A, FOS, APP, TICAM1, BCL3, TCF4, NR2F2, TRAF6, SYK, EGFR, IRAK1, SOX10, AR, TGFBR1, RELA, TP53BP1, RXRA, TRIM28, CREBBP, SMAD2, BRCA1, STAT3, HDAC4, MNAT1, SALL4, YWHAH, EP300, MAPK14, ETS2, UBC, PDGFRA, IRF1, JAK2, MTOR, PIAS1, SMARCA2, SMARCA4	1.71E-14
GO:0042981~regulation of apoptosis	HMGB1, YWHAZ, STAT5A, ERBB2, STAT5B, TLR2, TLR4, CHEK2, SRC, BTK, CTNNBL1, AKT1, VDR, BAK1, APP, CASP3, MYD88, RAC1, PRKRA, TICAM1, RHOA, BCL3, CASP1, TRAF6, DNAJA3, EGFR, IRAK1, IL2RB, ACTN4, DFFA, TGFBR1, RELA, RXRA, YWHAB, CIDEB, FADD, PRKCE, STAT1, BRCA1, TRADD, MNAT1, LCK, UBC, JAK2	6.80E-11
GO:0043066~negative regulation of apoptosis	EGFR, IRAK1, HMGB1, IL2RB, YWHAZ, DFFA, ERBB2, RELA, TGFBR1, STAT5A, STAT5B, AKT1, MNAT1, CASP3, MYD88, UBC, RHOA, BCL3, TRAF6, DNAJA3	1.95E-05
GO:0043067~regulation of programmed cell death	HMGB1, YWHAZ, STAT5A, ERBB2, STAT5B, TLR2, TLR4, CHEK2, KIT, SRC, BTK, CTNNBL1, AKT1, VDR, BAK1, APP, CASP3, MYD88, RAC1, PRKRA, TICAM1, RHOA, BCL3, CASP1, TRAF6, DNAJA3, EGFR, IRAK1, IL2RB, ACTN4, DFFA, TGFBR1, RELA, RXRA, YWHAB, CIDEB, FADD, PRKCE, STAT1, BRCA1, TRADD, MNAT1, LCK, UBC, JAK2	2.55E-11
GO:0043069~negative regulation of programmed cell death	EGFR, IRAK1, HMGB1, IL2RB, YWHAZ, DFFA, ERBB2, RELA, TGFBR1, STAT5A, STAT5B, KIT, AKT1, MNAT1, CASP3, MYD88, UBC, RHOA, BCL3, TRAF6, DNAJA3	6.72E-06
GO:0045941~positive regulation of transcription	E2F1, HMGB2, STAT5A, SOX2, STAT5B, SOX8, FOS, TNFRSF1A, APP, BCL3, TCF4, NR2F2, SOX10, IRAK1, AR, TGFBR1, TP53BP1, RXRA, RELA, CREBBP, TRIM28, SMAD2, BRCA1, STAT3, HDAC4, MNAT1, SALL4, YWHAH, EP300, MAPK14, ETS2, UBC, IRF1, PIAS1, SMARCA2, SMARCA4	9.79E-11
GO:0060548~negative regulation of cell death	EGFR, IRAK1, HMGB1, IL2RB, YWHAZ, DFFA, ERBB2, RELA, TGFBR1, STAT5A, STAT5B, KIT, AKT1, MNAT1, CASP3, MYD88, UBC, RHOA, BCL3, TRAF6, DNAJA3	7.04E-06

Table S1. *Cont*.

MIMAT0000449(hsa-miR-146a)		
**GO-term**	**Genes**	**P-value**
GO:0006916~anti-apoptosis	CDK1, IRAK1, MYD88, HDAC1, RELA, BAG3, STAT5A, UBC, SKP2, TNFAIP3, TRAF6, THBS1	1.14E-04
GO:0009891~positive regulation of biosynthetic process	E2F1, HRAS, STAT5A, TLR3, TLR4, CCNE1, TNFRSF1A, SMARCB1, TCF4, TRAF6, THBS1, PTX3, TCF3, CCNA2, LTB, CEBPA, IRAK1, TP53BP1, RELA, SMAD3, SMAD2, KAT5, BRCA1, CDK2, HDAC2, EP300, HDAC1, SP1, SMARCC1, SMARCC2, UBC, SMARCA2, SMARCA4	9.27E-10
GO:0010557~positive regulation of macromolecule biosynthetic process	E2F1, HRAS, STAT5A, TLR3, TLR4, CCNE1, TNFRSF1A, SMARCB1, TCF4, TRAF6, THBS1, TCF3, CCNA2, LTB, CEBPA, IRAK1, TP53BP1, RELA, SMAD3, SMAD2, KAT5, BRCA1, CDK2, HDAC2, EP300, HDAC1, SP1, SMARCC1, SMARCC2, UBC, SMARCA2, SMARCA4	8.75E-10
GO:0010604~positive regulation of macromolecule metabolic process	E2F1, HRAS, STAT5A, TLR3, TLR4, PF4, CCNE1, TNFRSF1A, SMARCB1, PSMD3, H2AFX, TCF4, PSMD6, THBS1, TRAF6, LTB, CCNA2, TCF3, TERF2, CEBPA, IRAK1, CDK1, BRCC3, RELA, TP53BP1, SMAD3, SMAD2, KAT5, BRCA1, CDK2, RAD51, RNF8, HDAC2, EP300, SP1, HDAC1, PLK1, SMARCC1, SMARCC2, UBC, IL12A, BRE, SMURF1, SMARCA2, BARD1, SMARCA4	1.28E-15
GO:0010605~negative regulation of macromolecule metabolic process	E2F1, PF4, TERF2IP, ZBTB16, VDR, IRAK3, PSMD3, TCF4, THBS1, PSMD6, TERF2, CEBPA, RELA, SKP2, SMAD3, BRCA2, SMAD2, TLE1, ATR, KAT5, SIRT1, BRCA1, SIRT2, FLNA, SIRT3, HDAC2, HDAC1, SMARCC2, UBC, BUB1B, SMARCA2, RAD17, BARD1, SMARCA4	8.91E-10
GO:0010628~positive regulation of gene expression	E2F1, STAT5A, PF4, TNFRSF1A, CCNE1, SMARCB1, TCF4, TCF3, CCNA2, CEBPA, IRAK1, TP53BP1, RELA, SMAD3, SMAD2, KAT5, CDK2, BRCA1, HDAC2, EP300, HDAC1, SP1, SMARCC1, SMARCC2, UBC, SMARCA2, SMARCA4	7.48E-08
GO:0010941~regulation of cell death	HRAS, MMP9, STAT5A, MGMT, TNFSF14, PF4, TLR4, ZBTB16, CHEK2, VDR, MYD88, BAG3, TRAF6, THBS1, LTB, LTA, CDK1, IRAK1, RELA, SKP2, SMAD3, BRCA2, SIRT1, ATM, BRCA1, SARM1, HDAC1, UBC, IL12A, TNFAIP3, ABL1, BARD1	1.47E-07
GO:0031328~positive regulation of cellular biosynthetic process	E2F1, HRAS, STAT5A, TLR3, TLR4, CCNE1, TNFRSF1A, SMARCB1, TCF4, TRAF6, THBS1, PTX3, TCF3, CCNA2, LTB, CEBPA, IRAK1, TP53BP1, RELA, SMAD3, SMAD2, KAT5, BRCA1, CDK2, HDAC2, EP300, HDAC1, SP1, SMARCC1, SMARCC2, UBC, SMARCA2, SMARCA4	6.46E-10
GO:0042981~regulation of apoptosis	HRAS, MMP9, STAT5A, MGMT, TNFSF14, PF4, TLR4, ZBTB16, CHEK2, VDR, MYD88, BAG3, TRAF6, THBS1, LTB, LTA, CDK1, IRAK1, RELA, SKP2, SMAD3, BRCA2, SIRT1, ATM, BRCA1, SARM1, HDAC1, UBC, IL12A, TNFAIP3, ABL1, BARD1	1.09E-07
GO:0043066~negative regulation of apoptosis	IRAK1, CDK1, HRAS, RELA, STAT5A, SKP2, SMAD3, PF4, ATM, MYD88, HDAC1, BAG3, UBC, TNFAIP3, TRAF6, THBS1, BARD1	2.51E-05
GO:0043067~regulation of programmed cell death	HRAS, MMP9, STAT5A, MGMT, TNFSF14, PF4, TLR4, ZBTB16, CHEK2, VDR, MYD88, BAG3, TRAF6, THBS1, LTB, LTA, CDK1, IRAK1, RELA, SKP2, SMAD3, BRCA2, SIRT1, ATM, BRCA1, SARM1, HDAC1, UBC, IL12A, TNFAIP3, ABL1, BARD1	1.36E-07
GO:0043069~negative regulation of programmed cell death	IRAK1, CDK1, HRAS, RELA, STAT5A, SKP2, SMAD3, PF4, ATM, MYD88, HDAC1, BAG3, UBC, TNFAIP3, TRAF6, THBS1, BARD1	2.98E-05
GO:0045941~positive regulation of transcription	E2F1, STAT5A, TNFRSF1A, CCNE1, SMARCB1, TCF4, TCF3, CCNA2, CEBPA, IRAK1, TP53BP1, RELA, SMAD3, SMAD2, KAT5, CDK2, BRCA1, HDAC2, EP300, HDAC1, SP1, SMARCC1, SMARCC2, UBC, SMARCA2, SMARCA4	1.64E-07
GO:0060548~negative regulation of cell death	IRAK1, CDK1, HRAS, RELA, STAT5A, SKP2, SMAD3, PF4, ATM, MYD88, HDAC1, BAG3, UBC, TNFAIP3, TRAF6, THBS1, BARD1	3.08E-05

Table S1. *Cont.*

MIMAT0000267(hsa-miR-210)		
**GO-term**	**Genes**	**P-value**
GO:0006916~anti-apoptosis	HMGB1, HDAC1, UBC, MYC, DAPK1	1.43E-04
GO:0009891~positive regulation of biosynthetic process	CEBPA, HDAC1, UBC, ELK1, SIRT7, FGF2, MYC	0.00517
GO:0010557~positive regulation of macromolecule biosynthetic process	CEBPA, HDAC1, UBC, ELK1, SIRT7, FGF2, MYC	0.0293
GO:0010604~positive regulation of macromolecule metabolic process	UBE2N, CEBPA, HDAC1, UBC, ELK1, SIRT7, UBE2D1, FGF2, MYC	0.022541
GO:0010605~negative regulation of macromolecule metabolic process	CEBPA, HMGB1, SIN3A, HDAC1, MLX, YWHAB, UBC, SIRT7, UBE2D1, FGF2, MYC	0.00764
GO:0010628~positive regulation of gene expression	CEBPA, HDAC1, UBC, ELK1, SIRT7, FGF2, MYC	0.013297
GO:0010941~regulation of cell death	HMGB1, HDAC1, YWHAB, UBC, MNT, SFN, FGF2, MYC, DAPK1	0.005681
GO:0031328~positive regulation of cellular biosynthetic process	CEBPA, HDAC1, UBC, ELK1, SIRT7, FGF2, MYC	0.027541
GO:0042981~regulation of apoptosis	HMGB1, HDAC1, YWHAB, UBC, MNT, SFN, MYC, DAPK1	0.017988
GO:0043066~negative regulation of apoptosis	HMGB1, HDAC1, UBC, MYC, DAPK1	0.031761
GO:0043067~regulation of programmed cell death	HMGB1, HDAC1, YWHAB, UBC, MNT, SFN, FGF2, MYC, DAPK1	0.005558
GO:0043069~negative regulation of programmed cell death	HMGB1, HDAC1, UBC, MYC, DAPK1	0.033195
GO:0045941~positive regulation of transcription	CEBPA, HDAC1, UBC, ELK1, SIRT7, FGF2, MYC	0.011608
GO:0060548~negative regulation of cell death	HMGB1, HDAC1, UBC, MYC, DAPK1	0.033486

Table S1. *Cont.*

MIMAT0000762(hsa-miR-324-3p)		
**GO-term**	**Genes**	**P-value**
GO:0006916~anti-apoptosis	PEA15, YWHAZ, NPM1, UBC, ESR1, ESR2, PRNP, CASP2	0.001972
GO:0009891~positive regulation of biosynthetic process	E2F1, PPARA, THRB, RXRA, CASK, RB1, SIRT7, ESR2, SHH, ATXN1, HDAC5, NCOA1, NPM1, NCOA6, UBC, CAND1	8.64E-04
GO:0010557~positive regulation of macromolecule biosynthetic process	E2F1, PPARA, THRB, RXRA, CASK, RB1, ESR2, SIRT7, SHH, ATXN1, HDAC5, NCOA1, NCOA6, UBC, CAND1	0.001401
GO:0010604~positive regulation of macromolecule metabolic process	E2F1, PPARA, THRB, RXRA, ESR1, CASK, RB1, SIRT7, ESR2, SHH, ATXN1, HDAC5, NCOA1, PSMB3, NCOA6, UBC, CAND1	0.002607
GO:0010605~negative regulation of macromolecule metabolic process	E2F1, PPARA, THRB, TSG101, RXRA, RB1, SIRT7, ATR, SHH, ATXN1, HDAC5, EIF4A3, ITGAV, PSMB3, NPM1, UBC, ADAR	5.16E-04
GO:0010628~positive regulation of gene expression	E2F1, PPARA, THRB, RXRA, ESR1, CASK, RB1, SIRT7, ESR2, SHH, ATXN1, HDAC5, NCOA1, NCOA6, UBC, CAND1	1.29E-04
GO:0010941~regulation of cell death	YWHAZ, RXRA, MGMT, ESR1, ESR2, SHH, CUL3, PEA15, CUL5, GSPT1, ILK, NPM1, UBC, PRNP, CASP2	0.009867
GO:0031328~positive regulation of cellular biosynthetic process	E2F1, PPARA, THRB, RXRA, CASK, RB1, SIRT7, ESR2, SHH, ATXN1, HDAC5, NCOA1, NPM1, NCOA6, UBC, CAND1	7.46E-04
GO:0042981~regulation of apoptosis	YWHAZ, RXRA, MGMT, ESR1, ESR2, SHH, CUL3, PEA15, CUL5, GSPT1, ILK, NPM1, UBC, PRNP, CASP2	0.008815
GO:0043066~negative regulation of apoptosis	PEA15, YWHAZ, ILK, NPM1, UBC, ESR1, ESR2, PRNP, CASP2, SHH	0.003395
GO:0043067~regulation of programmed cell death	YWHAZ, RXRA, MGMT, ESR1, ESR2, SHH, CUL3, PEA15, CUL5, GSPT1, ILK, NPM1, UBC, PRNP, CASP2	0.009571
GO:0043069~negative regulation of programmed cell death	PEA15, YWHAZ, ILK, NPM1, UBC, ESR1, ESR2, PRNP, CASP2, SHH	0.003724
GO:0045941~positive regulation of transcription	E2F1, PPARA, THRB, RXRA, CASK, RB1, ESR2, SIRT7, SHH, ATXN1, HDAC5, NCOA1, NCOA6, UBC, CAND1	3.29E-04
GO:0060548~negative regulation of cell death	PEA15, YWHAZ, ILK, NPM1, UBC, ESR1, ESR2, PRNP, CASP2, SHH	0.003792

Table S1. *Cont.*

MIMAT0004692(hsa-miR-340)		
**GO-term**	**Genes**	**P-value**
GO:0006916~anti-apoptosis	AKT1, IRAK1, XIAP, SOCS3, UBC, NFKBIA, RIPK2, MALT1, TRAF6, IKBKB, RPS27A	1.36E-05
GO:0009891~positive regulation of biosynthetic process	IRAK1, BCL10, ELANE, NFKBIA, TLR4, SIRT7, AKT1, CARD11, PRKCQ, TNFRSF1A, PKNOX1, ETS1, F2, UBC, BCL3, CAND1, TRAF6, NFIC, RPS27A, TLX1	9.68E-06
GO:0010557~positive regulation of macromolecule biosynthetic process	IRAK1, BCL10, ELANE, NFKBIA, TLR4, SIRT7, AKT1, CARD11, PRKCQ, TNFRSF1A, PKNOX1, ETS1, F2, UBC, BCL3, CAND1, TRAF6, NFIC, RPS27A, TLX1	4.05E-06
GO:0010604~positive regulation of macromolecule metabolic process	BTRC, NFKBIA, TLR4, PSMA7, AKT1, TNFRSF1A, PSMD3, BCL3, PSMD4, PSMD6, TRAF6, PSMD7, CUL1, TLX1, RPS27A, IRAK1, BCL10, ELANE, SKP1, SIRT7, UBE2N, PSMA2, CARD11, GH1, PRKCQ, PSMD14, PSMC6, CARD14, PSMD13, PKNOX1, PSMD12, ETS1, PSMD10, F2, UBC, CAND1, NFIC	8.50E-16
GO:0010605~negative regulation of macromolecule metabolic process	ELANE, SIRT7, PSMA7, SIRT2, PSMA2, SUMO1, PSMC6, PSMD14, PSMD13, PSMD12, PSMD10, PPP2CA, EIF3E, UBC, PSMD3, BCL3, PSMD4, PSMD6, NFIC, PSMD7, RPS27A	5.74E-06
GO:0010628~positive regulation of gene expression	IRAK1, BCL10, NFKBIA, SIRT7, TNFRSF1A, PKNOX1, ETS1, UBC, BCL3, CAND1, NFIC, RPS27A, TLX1	0.005076
GO:0010941~regulation of cell death	TRAF2, XIAP, NFKBIA, TLR4, CUL3, AKT1, MAP3K7, NLRC4, CUL5, CASP9, PPP2CA, PPP2CB, BCL3, TRAF6, CUL1, RPS27A, IRAK1, BCL10, CARD9, SOCS3, MALT1, BIRC2, PLG, CARD10, TRADD, TNFRSF10A, CARD11, NME2, CARD14, CAPN10, ETS1, F2, IKBKG, UBC, RIPK2, IKBKB	1.29E-15
GO:0031328~positive regulation of cellular biosynthetic process	IRAK1, BCL10, ELANE, NFKBIA, TLR4, SIRT7, AKT1, CARD11, PRKCQ, TNFRSF1A, PKNOX1, ETS1, UBC, BCL3, CAND1, TRAF6, NFIC, RPS27A, TLX1	2.89E-05
GO:0042981~regulation of apoptosis	TRAF2, XIAP, NFKBIA, TLR4, CUL3, AKT1, MAP3K7, NLRC4, CUL5, CASP9, PPP2CA, PPP2CB, BCL3, TRAF6, CUL1, RPS27A, IRAK1, BCL10, CARD9, SOCS3, MALT1, BIRC2, PLG, CARD10, TRADD, TNFRSF10A, CARD11, NME2, CARD14, CAPN10, ETS1, F2, IKBKG, UBC, RIPK2, IKBKB	8.15E-16
GO:0043066~negative regulation of apoptosis	IRAK1, BCL10, XIAP, SOCS3, NFKBIA, MALT1, AKT1, MAP3K7, NME2, PPP2CB, UBC, RIPK2, BCL3, TRAF6, IKBKB, RPS27A	4.82E-07
GO:0043067~regulation of programmed cell death	TRAF2, XIAP, NFKBIA, TLR4, CUL3, AKT1, MAP3K7, NLRC4, CUL5, CASP9, PPP2CA, PPP2CB, BCL3, TRAF6, CUL1, RPS27A, IRAK1, BCL10, CARD9, SOCS3, MALT1, BIRC2, PLG, CARD10, TRADD, TNFRSF10A, CARD11, NME2, CARD14, CAPN10, ETS1, F2, IKBKG, UBC, RIPK2, IKBKB	1.06E-15
GO:0043069~negative regulation of programmed cell death	IRAK1, BCL10, XIAP, SOCS3, NFKBIA, MALT1, AKT1, MAP3K7, NME2, PPP2CB, UBC, RIPK2, BCL3, TRAF6, IKBKB, RPS27A	5.76E-07
GO:0045941~positive regulation of transcription	IRAK1, BCL10, NFKBIA, SIRT7, TNFRSF1A, PKNOX1, ETS1, UBC, BCL3, CAND1, NFIC, RPS27A, TLX1	0.004015
GO:0060548~negative regulation of cell death	IRAK1, BCL10, XIAP, SOCS3, NFKBIA, MALT1, AKT1, MAP3K7, NME2, PPP2CB, UBC, RIPK2, BCL3, TRAF6, IKBKB, RPS27A	5.97E-07

Table S1. *Cont.*

MIMAT0002174(hsa-miR-484)		
**GO-term**	**Genes**	**P-value**
GO:0006916~anti-apoptosis	YWHAZ, SQSTM1, CRYAB, UBC, TGM2, IGF1, TRAF6, SOD1, THBS1, UBA52	0.009451
GO:0009891~positive regulation of biosynthetic process	MYOD1, HRAS, NIF3L1, GRIP1, PAX3, CTNNB1, SQSTM1, TRAF6, THBS1, TCF3, EGFR, SMAD4, ITGA2, IGF1, SMAD2, IGF2, ATXN7L3, TNNI2, SREBF2, HDAC4, PSMC5, SP1, ATXN7, UBC, UBA52	9.23E-04
GO:0010557~positive regulation of macromolecule biosynthetic process	MYOD1, HRAS, NIF3L1, GRIP1, SMAD4, ITGA2, IGF1, IGF2, SMAD2, PAX3, ATXN7L3, TNNI2, CTNNB1, SREBF2, HDAC4, PSMC5, SP1, SQSTM1, ATXN7, UBC, TRAF6, THBS1, TCF3, UBA52	9.30E-04
GO:0010604~positive regulation of macromolecule metabolic process	MYOD1, HRAS, NIF3L1, GRIP1, BTRC, PAX3, CTNNB1, PSMD1, PSMD2, PSMD3, PSMD4, PSMD5, PSMD6, PSMD7, CUL1, PSMD8, PRKCA, F12, PRKCG, UBE2C, PSMA2, PSMA1, PSMA6, PSMA5, PSMA3, UBC, MDM2, PSME3, UBA52, CAV1, PSMA7, STUB1, PSMB7, PSMB1, SQSTM1, TRAF6, THBS1, TCF3, TINF2, APC, SMAD4, IGF1, ITGA2, SMAD2, IGF2, FURIN, ATXN7L3, TNNI2, SREBF2, CCNB1, HDAC4, PSMC6, PSMD14, PSMC5, PSMD13, PSMD12, SP1, PSMC4, PSMC3, NEDD4, PSMD11, PSMC2, ATXN7, PSMD10, PSMC1	1.25E-24
GO:0010605~negative regulation of macromolecule metabolic process	COPS2, PAX3, CTNNB1, GFI1B, PSMD1, PSMD2, PSMD3, PSMD4, PSMD5, PSMD6, PSMD7, PSMD8, PRKCA, PRKCG, TLE1, UBE2C, PSMA2, PSMA1, PSMA6, PSMA5, PSMA3, UBC, MDM2, PSME3, UBA52, EIF2C4, CAV1, PSMA7, TIMP3, PSMB7, PSMB1, ITGAV, GFI1, THBS1, TINF2, MSH6, YWHAB, SMAD4, IGF2, SMAD2, FURIN, YWHAE, HDAC4, PSMC6, PSMD14, PSMD13, PSMC5, PSMD12, PSMC4, PSMC3, NEDD4, ID1, PSMD11, PSMD10, PSMC2, PSMC1	4.03E-21
GO:0010628~positive regulation of gene expression	MYOD1, NIF3L1, GRIP1, SMAD4, IGF1, SMAD2, PAX3, ATXN7L3, TNNI2, SREBF2, CTNNB1, HDAC4, PSMC5, SP1, SQSTM1, ATXN7, UBC, TCF3, UBA52	0.011776
GO:0010941~regulation of cell death	YWHAZ, HRAS, ERBB3, GRIK2, ERBB2, BCAR1, TIMP3, SRC, MAGED1, CUL3, CUL2, ALB, SQSTM1, TGM2, THBS1, TRAF6, CUL1, APC, EGFR, PRKCA, MSH6, CRYAB, YWHAB, IGF1, IGF2, SOD1, FURIN, YWHAE, PSMC5, BTG2, UBC, PSME3, ABL1, UBA52	3.89E-06
GO:0031328~positive regulation of cellular biosynthetic process	MYOD1, HRAS, NIF3L1, GRIP1, PAX3, CTNNB1, SQSTM1, TRAF6, THBS1, TCF3, EGFR, SMAD4, ITGA2, IGF1, SMAD2, IGF2, ATXN7L3, TNNI2, SREBF2, HDAC4, PSMC5, SP1, ATXN7, UBC, UBA52	7.55E-04
GO:0042981~regulation of apoptosis	YWHAZ, HRAS, ERBB3, GRIK2, ERBB2, BCAR1, TIMP3, SRC, MAGED1, CUL3, CUL2, ALB, SQSTM1, TGM2, THBS1, TRAF6, CUL1, APC, EGFR, PRKCA, MSH6, CRYAB, YWHAB, IGF1, IGF2, SOD1, FURIN, YWHAE, BTG2, UBC, PSME3, ABL1, UBA52	7.89E-06
GO:0043066~negative regulation of apoptosis	EGFR, HRAS, YWHAZ, ERBB3, GRIK2, CRYAB, ERBB2, IGF1, IGF2, SOD1, FURIN, BTG2, SQSTM1, ALB, UBC, TGM2, TRAF6, THBS1, UBA52, APC	1.21E-05
GO:0043067~regulation of programmed cell death	YWHAZ, HRAS, ERBB3, GRIK2, ERBB2, BCAR1, TIMP3, SRC, MAGED1, CUL3, CUL2, ALB, SQSTM1, TGM2, THBS1, TRAF6, CUL1, APC, EGFR, PRKCA, MSH6, CRYAB, YWHAB, IGF1, IGF2, SOD1, FURIN, YWHAE, PSMC5, BTG2, UBC, PSME3, ABL1, UBA52	3.59E-06
GO:0043069~negative regulation of programmed cell death	EGFR, HRAS, YWHAZ, ERBB3, GRIK2, CRYAB, ERBB2, IGF1, IGF2, SOD1, FURIN, PSMC5, BTG2, SQSTM1, ALB, UBC, TGM2, TRAF6, THBS1, UBA52, APC	4.03E-06
GO:0045941~positive regulation of transcription	MYOD1, NIF3L1, GRIP1, SMAD4, IGF1, SMAD2, PAX3, ATXN7L3, TNNI2, SREBF2, CTNNB1, HDAC4, PSMC5, SP1, SQSTM1, ATXN7, UBC, TCF3, UBA52	0.008885
GO:0060548~negative regulation of cell death	EGFR, HRAS, YWHAZ, ERBB3, GRIK2, CRYAB, ERBB2, IGF1, IGF2, SOD1, FURIN, PSMC5, BTG2, SQSTM1, ALB, UBC, TGM2, TRAF6, THBS1, UBA52, APC	4.23E-06

Table S1. *Cont.*

MIMAT0002177(hsa-miR-486-5p)		
**GO-term**	**Genes**	**P-value**
GO:0006916~anti-apoptosis	YWHAZ, GNRH1, GSK3B, UBC, THBS1	0.00119
GO:0009891~positive regulation of biosynthetic process	WNT1, YWHAH, SMAD9, UBC, THBS1, CTNNB1	0.01975
GO:0010557~positive regulation of macromolecule biosynthetic process	WNT1, YWHAH, SMAD9, UBC, THBS1, CTNNB1	0.015555
GO:0010604~positive regulation of macromolecule metabolic process	WNT1, YWHAH, SMAD9, UBC, THBS1, CTNNB1, AXIN1	0.011952
GO:0010605~negative regulation of macromolecule metabolic process	YWHAB, YWHAQ, UBC, THBS1, YWHAE, CTNNB1	0.024386
GO:0010628~positive regulation of gene expression	WNT1, YWHAH, SMAD9, UBC, CTNNB1	0.042287
GO:0010941~regulation of cell death	YWHAZ, GNRH1, GSK3B, YWHAB, UBC, SFN, ABL1, THBS1, YWHAE	3.82E-04
GO:0031328~positive regulation of cellular biosynthetic process	WNT1, YWHAH, SMAD9, UBC, THBS1, CTNNB1	0.018664
GO:0042981~regulation of apoptosis	YWHAZ, GNRH1, GSK3B, YWHAB, UBC, SFN, ABL1, THBS1, YWHAE	3.48E-04
GO:0043066~negative regulation of apoptosis	YWHAZ, GNRH1, GSK3B, UBC, THBS1	0.008301
GO:0043067~regulation of programmed cell death	YWHAZ, GNRH1, GSK3B, YWHAB, UBC, SFN, ABL1, THBS1, YWHAE	3.73E-04
GO:0043069~negative regulation of programmed cell death	YWHAZ, GNRH1, GSK3B, UBC, THBS1	0.008713
GO:0045941~positive regulation of transcription	WNT1, YWHAH, SMAD9, UBC, CTNNB1	0.038563
GO:0060548~negative regulation of cell death	YWHAZ, GNRH1, GSK3B, UBC, THBS1	0.008797
